# The diagnostic journey of fibrodysplasia ossificans progressiva: evidence from a national cohort of 114 patients

**DOI:** 10.3389/fendo.2026.1941326

**Published:** 2026-10-14

**Authors:** Lijia Cui, Quanshi Ouyang, Jian Guo, Wanping Cai, Qianqian Pang, Yue Chi, Ruizhi Jiajue, Xiang Li, Wei Liu, Yan Jiang, Ou Wang, Mei Li, Xiaoping Xing, Qiming Xu, Weibo Xia

**Affiliations:** 1 Department of Endocrinology, National Health Commission (NHC) Key Laboratory of Endocrinology, Peking Union Medical College Hospital, Chinese Academy of Medical Sciences & Peking Union Medical College, Beijing, China; 2 State Key Laboratory of Complex, Severe, and Rare Diseases, Institute of Clinical Medicine, Peking Union Medical College Hospital, Chinese Academy of Medical Sciences & Peking Union Medical College, Beijing, China; 3 Department of Orthopedic Oncology, Beijing Jishuitan Hospital, Capital Medical University, Beijing, China

**Keywords:** Acvr1, diagnostic delay, fibrodysplasia ossificans progressiva, iatrogenic harm, misdiagnosis, missed diagnosis

## Abstract

**Background:**

Fibrodysplasia ossificans progressiva (FOP) is an ultra-rare genetic disorder caused by gain-of-function variants in *ACVR1*, characterized by congenital great-toe malformations, recurrent inflammatory soft-tissue swellings, and progressive heterotopic ossification (HO). Early lesions may resemble neoplastic, inflammatory, infectious, or trauma-related conditions, whereas invasive procedures can trigger additional HO.

**Methods:**

We performed a cross-sectional analysis of 114 patients with molecularly confirmed FOP enrolled in the National Rare Diseases Registry System of China. The diagnostic journey was reconstructed to evaluate diagnostic delay, misdiagnosis, missed diagnostic opportunities, and patient-reported outcomes after high-risk procedures.

**Results:**

Patients were recruited from 23 provinces or regions. Diagnostic delay exceeded 3 years in 34.5% patients and 10 years in 9.7% patients. FOP was not considered at the first hospital visit in 85 of 114 patients (74.6%), and 72 of 114 (63.2%) reported misdiagnosis. Among misdiagnosed patients, tumor-related diagnoses were most common (36/72, 50.0%), followed by orthopedic or congenital (13/72, 18.1%) and inflammatory or infectious diagnoses (12/72, 16.7%). 102 of 114 patients (89.5%) reported congenital great-toe malformation. Biopsy was reported by 51 patients (44.7%), of whom 41 (80.4%) reported subsequent worsening; 22 of 25 patients (88.0%) who underwent HO excision reported recurrence or new lesions at the surgical site.

**Conclusion:**

Diagnostic errors remained common in FOP. Biopsy and HO excision were also frequently reported in FOP patients and were often followed by patient-reported worsening or recurrence. These findings provide a rationale for prospective evaluation of an early-recognition pathway emphasizing examination of the great toes, avoidance of invasive procedures when FOP is suspected, *ACVR1* testing, and specialist referral.

## Introduction

Fibrodysplasia ossificans progressiva (FOP) is an ultra-rare and severely disabling genetic disorder, with population-based prevalence estimates ranging from approximately 0.36 to 1.36 per million in European and North American populations (1–3). Its classic phenotype combines congenital skeletal malformations, particularly shortened and malformed great toes, with episodic formation of heterotopic bone in skeletal muscle, fascia, tendons, ligaments, and other connective tissues (4, 5). Postnatal disease activity often begins in childhood as painful or painless soft-tissue swellings and progresses through recurrent flare-ups and cumulative heterotopic ossification (HO), resulting in joint ankylosis, thoracic restriction, and substantial lifelong disability (6, 7). FOP is caused by heterozygous gain-of-function variants in *ACVR1* (also known as *ALK2*), which encodes a type I bone morphogenetic protein receptor (8, 9). In addition to dysregulated bone morphogenetic protein signaling, the mutant receptor acquires abnormal responsiveness to activin A, providing a mechanistic link between inflammatory episodes and heterotopic bone formation (10).

The congenital great-toe phenotype precedes postnatal HO and therefore represents an early, non-invasive diagnostic clue (11–13). The combination of malformed great toes and compatible soft-tissue or radiographic findings (typical anatomic distribution and maturation of HO) can raise clinical suspicion of FOP (14, 15). Molecular testing for *ACVR1* can confirm the diagnosis, particularly in early, atypical, or clinically uncertain presentations.

Despite these distinctive features, FOP remains highly susceptible to diagnostic error (14). Early flare-ups may mimic sarcoma, aggressive fibromatosis, infection, lymphadenitis, myositis, traumatic hematoma, or other orthopedic and inflammatory disorders. Histologically, early lesions can be highly cellular and may be mistaken for a neoplastic process (16, 17). This diagnostic uncertainty is clinically consequential because biopsy, surgical excision, intramuscular injection, mandibular block, and forceful manipulation can provoke flare-ups and additional HO (17). Recognition of FOP is therefore not only a diagnostic challenge but also a patient-safety priority.

Previous studies have described the clinical spectrum, genotype, natural history, and global diagnostic burden of FOP (1–3, 6, 7, 18–21). However, national data focused specifically on the diagnostic journey of patients with FOP remain limited. In particular, misdiagnosis—the assignment of an incorrect disease label—and a missed diagnostic opportunity—the failure to connect available clinical clues—have seldom been evaluated separately.

In this national cohort of 114 patients with FOP, we aimed to characterize the pathway from the first disease-related symptom to correct diagnosis. We quantified diagnostic delay, described the specialties involved and the patterns of misdiagnosis, examined patient-reported outcomes after high-risk diagnostic and therapeutic procedures, and developed a pragmatic early-recognition pathway intended for clinicians who may encounter FOP before referral to a specialist center.

## Materials and methods

### Study design and setting

We conducted a cross-sectional analysis of patients with FOP, registered in National Rare Diseases Registry System of China (22). Registry information was collected using a structured questionnaire covering demographic characteristics, congenital skeletal features, current clinical manifestations, diagnostic history, previous misdiagnoses, investigations, and procedure-associated outcomes (**Supplementary Information**). Patients were recruited through the National Rare Diseases Registry System of China between April 2026 and June 2026, with the updated analytic export dated 12 June 2026. The questionnaire was completed by participants themselves when possible or by legal guardians for participants unable to complete it independently. Respondent type was not retained as a separate variable in the analytic export. The manuscript was prepared in accordance with the principles of the STROBE statement for observational studies (23).

### Study population

Patients were eligible if they had a reported diagnosis of FOP supported by typical clinical features, including characteristic progressive HO with/without congenital great-toe malformation, and genetic testing for confirmed *ACVR1* pathogenic mutation. The updated analytic dataset contained 114 patients with available questionnaire-level data. Genetic testing was performed using whole-exome sequencing in 91 patients (79.8%) and targeted Sanger sequencing of *ACVR1* in 23 patients (20.2%). Genetic testing reports were uploaded by participants. *ACVR1* variant descriptions from different laboratory reports were normalized to a single reference transcript NM_001105. Molecular eligibility was based on an *ACVR1* variant reported as pathogenic or likely pathogenic by the clinical testing laboratory. This study was approved by the local ethics committee of Peking Union Medical College Hospital (PUMCH) (Ethics approval number: I-25PJ2872) and informed consent was obtained from all participants or their legal guardians.

### Data collection

Collected data included demographic characteristics (sex and current age); diagnostic journey variables (age at the first FOP-related symptom, age at the first hospital visit, age at definitive FOP diagnosis, first-contact hospital department, department giving the first FOP diagnosis, reported diagnostic delay, misdiagnosed diseases, and high-risk events and procedures); genetic testing for *ACVR1*; disease-related clinical features (congenital skeletal features, HO sites, affected joints); and functional outcomes (quality-of-life score). Participants were grouped as < 12 years, 12–18 years, and > 18 years to represent younger childhood, adolescence, and adulthood, respectively. Self-reported quality of life was assessed on a scale from 0 to 10, with higher scores indicating better quality of life (24). The number of affected joints with restricted movement was calculated by assigning one point to each of 16 prespecified sites: the temporomandibular joint; cervical, thoracic and lumbar spine; and the left and right shoulder, elbow, metacarpophalangeal, hip, knee, and ankle joints.

### Variable coding

Because the dataset was derived from a structured questionnaire, variables based on free text were cleaned and coded according to predefined rules. Hospital and department names were standardized; reported diagnoses and symptoms were mapped to clinical categories; anatomical sites were coded by body region, with laterality recorded when specified; and HO size was classified as <2 cm, 2–5 cm, or >5 cm. Genetic results were standardized to HGVS nomenclature, and multiple comorbidities were recorded separately. “Other” responses were reassigned to existing categories where appropriate. Numeric entries were checked, and implausible values were verified against the original questionnaire or treated as missing. Ages and time intervals entered as free text were normalized to years. Expressions such as “half a year” were coded as 0.5 years, month-based expressions were divided by 12, and approximate ranges were coded using their midpoint. Expressions indicating less than 1 year or less than 1 month were coded as 0.5 years and 0.5 months, respectively. Responses such as “unknown”, “not sure”, “skipped”, or unparseable free text were treated as missing.

### Variable definitions

Correct diagnosis was defined as the first reported diagnosis of FOP. First FOP-related symptom was defined as the earliest postnatal symptom recognized by the patient or caregiver as compatible with FOP (e.g. local mass or swelling, joint mobility restriction), excluding congenital great-toe malformation. Responses reporting an onset age of 0 years were reviewed together with the corresponding free-text description. When the description referred exclusively to a congenital skeletal feature, such as congenital finger or toe malformation, the age at first postnatal FOP-related manifestation was coded as missing. An onset age of 0 years was retained when the description indicated a noncongenital clinical manifestation occurring during the first year of life. Diagnostic delay was defined as the reported interval between first FOP-related symptom and correct FOP diagnosis. Two additional intervals were calculated at the patient level: symptom-to-first-visit (age at first hospital visit minus age at first FOP-related symptom) and first-visit-to-diagnosis (age at correct FOP diagnosis minus age at first hospital visit). Component diagnostic intervals were calculated only when both required ages were available and were in the expected chronological order. Records in which the first-visit age preceded the reported postnatal symptom-onset age, or the diagnosis age preceded the first-visit age, were treated as missing for the corresponding interval only; reported ages were not imputed or reordered. Misdiagnosis was defined as a patient-reported incorrect diagnosis before FOP recognition. The great-toe indicator was a questionnaire-derived proxy rather than an independently adjudicated assessment of clinician recognition.

### Outcomes

The primary outcome was reported diagnostic delay, defined both continuously in years and descriptively across analysis-defined duration categories. The secondary outcomes included the indicators when FOP was first suspected, first-contact specialties, specialties issuing first FOP diagnosis, proportion of patients reporting misdiagnosis, categories of incorrect diagnosis, and biopsy- and surgery-related worsening. The reported misdiagnoses were assigned to the following non-mutually exclusive category: tumor-related, inflammatory or infectious, orthopedic or congenital, neurologic or neuromuscular, rheumatologic or autoimmune, trauma-related, or other or unclear. High-risk diagnostic and therapeutic exposures included bone or soft-tissue biopsy and surgical excision of HO. Procedure exposures and outcomes were derived from separate questionnaire items (**Supplementary Information**). Biopsy exposure was defined by selecting “bone/soft-tissue biopsy” in item III.8. Item III.9 asked whether the biopsy caused worsening of the condition or an acute flare-up. Responses of “yes” and “no” were coded as reported post-biopsy worsening and no reported worsening, respectively. HO excision was identified from items VIII.4–VIII.5, which recorded whether an HO lesion had been surgically excised and, where applicable, the surgical site, date, and postoperative recurrence or development of new HO at the surgical site. A report of recurrence or new HO at the operative site was coded as positive; an explicit report of no recurrence was coded as negative; and blank or indeterminate responses were treated as missing. A post-procedure worsening was reviewed by the researchers based on the details participants provided; this review did not constitute independent clinical confirmation of the reported outcomes. Because the timing of individual misdiagnosis episodes and procedures was not systematically recorded, no temporal or causal relationship between diagnostic error, procedural exposure, and subsequent outcomes was inferred.

### Statistical analysis

Continuous variables were summarized as median and interquartile range (IQR). Categorical variables were summarized as count and percentage. Diagnostic delay was grouped as ≤1 year, >1–3 years, >3–5 years, >5–10 years, or >10 years. The categories distinguish diagnosis within 1 year, the 3-year benchmark supported by a previous Chinese FOP survey (21), progressively longer delays, and delays exceeding 10 years. Five responses regarding total diagnostic delay were bounded or approximate: two responses of “less than 1 year,” one of “less than 1 month,” one of “1 year plus,” and one of “5 years plus.” For the primary analysis, these responses were assigned midpoint values of 0.5 year, 0.5/12 year, 1.5 years, and 5.5 years, respectively. Sensitivity analyses were performed using the lower and upper bounds of each interval. The lower-bound scenario assigned values of 0, 0, 1, and 5 years, whereas the upper-bound scenario assigned values of 1, 1/12, 2, and 6 years, respectively. All analyses were performed using R version 4.6.0 with the readxl, dplyr, tidyr, stringr, purrr, ggplot2, broom, writexl, patchwork, sf, tibble, ggrepel, and ggsci packages.

## Results

### Baseline characteristics

A total of 154 patients were assessed for a diagnosis of FOP and have basic information registered. 37 patients were excluded because they didn’t have questionnaire data submitted. Three patients were excluded because their questionnaire data were ambiguous or incomplete, leaving 114 patients in the final analysis (**Supplementary Figure 1**). Baseline characteristics of 114 FOP are shown in **Table 1**. Patients came from 23 provinces or regions in China (**Supplementary Figure 2**). Fifty-nine patients were male (51.8%) and 55 were female (48.2%). The median current age was 14.5 (9.0–23.5) years. The median age at first FOP-related symptom was 3.0 (1.1–6.0) years. Congenital great-toe malformation was reported by 102/114 (89.5%), cervical spine fusion or limited neck motion by 75/114 (65.8%). Current HO was reported by 105/114 (92.1%), and joint mobility restriction by 111/114 (97.4%). The median self-rated quality-of-life score was 6.0 (4.0–7.6). 97.4% (111/114) of the patients carried *ACVR1* [NM_001105]: c.617G>A (p.Arg206His) mutation, while the remaining 3 patients carried *ACVR1* [NM_001105]: c.982G>T (p.G328W) mutation.

**Table 1:** Baseline demographic and clinical characteristics of 114 patients with FOP.

Characteristic	Overall (n=114)	<12 years (n=45)	12–18 years (n=26)	>18 years (n=43)
Male sex, n/N (%)	59/114 (51.8)	23/45 (51.1)	14/26 (53.8)	22/43 (51.2)
Current age, median years (IQR)	14.5 (9.0–23.5); n=114	8.0 (5.0–10.0); n=45	15.0 (13.0–15.8); n=26	28.0 (21.5–33.5); n=43
Age at first FOP-related symptom, median years (IQR)	3.0 (1.2–6.0); n=111	2.0 (1.0–4.0); n=45	3.5 (1.5–6.0); n=25	3.0 (2.0–7.0); n=41
Age at first hospital visit, median years (IQR)	4.0 (2.0–7.0); n=112	2.4 (1.3–5.0); n=44	4.5 (2.2–7.5); n=26	6.0 (3.0–12.0); n=42
Age at correct FOP diagnosis, median years (IQR)	5.9 (3.0–11.0); n=113	3.5 (2.0–5.9); n=45	5.0 (3.6–10.0); n=26	11.5 (6.0–17.0); n=42
Congenital great-toe malformation, n/N (%)	102/114 (89.5)	41/45 (91.1)	25/26 (96.2)	36/43 (83.7)
Cervical spine fusion/limited neck motion, n/N (%)	75/114 (65.8)	28/45 (62.2)	15/26 (57.7)	32/43 (74.4)
Heterotopic ossification currently present, n/N (%)	105/114 (92.1)	40/45 (88.9)	26/26 (100.0)	39/43 (90.7)
Joint mobility restriction currently present, n/N (%)	111/114 (97.4)	42/45 (93.3)	26/26 (100.0)	43/43 (100.0)
Quality-of-life score, median (IQR)	6.0 (4.0–7.6); n=112	7.0 (5.0–8.0); n=45	5.0 (3.0–6.0); n=25	5.0 (3.0–6.0); n=42
Number of affected joints, median (IQR)	8.0 (5.0–12.5); n=111	5.5 (3.0–8.0); n=42	7.5 (5.0–11.5); n=26	13.0 (7.5–16.0); n=43

First FOP-related symptoms exclude congenital great-toe malformation. Self-reported quality of life was assessed on a scale from 0 to 10, with higher scores indicating better quality of life. The number of affected joints with restricted movement was calculated by assigning one point to each of 16 prespecified sites: the temporomandibular joint; cervical, thoracic and lumbar spine; and the left and right shoulder, elbow, metacarpophalangeal, hip, knee, and ankle joints.

Representative clinical and radiographic manifestations of FOP patients in this study are shown in **Figure 1**. Patients exhibited marked spinal deformities (**Figures 1A, B**), thoracic cage asymmetry (**Figure 1C**), and extensive HO involving the shoulder, extremity, and gluteal regions (**Figures 1D–F**). Congenital shortening and lateral deviation of the great toes, a characteristic early feature of FOP, were also observed (**Figures 1G, H**). **Figures 1I, J** further illustrates procedure-associated harm in a patient who underwent inappropriate surgical excision of HO around the left knee. The procedure was followed by the development of new and more extensive HO at the surgical site and complete immobility of the left knee joint, highlighting the risk of disease exacerbation after invasive intervention in patients with FOP.

**Figure 1:**
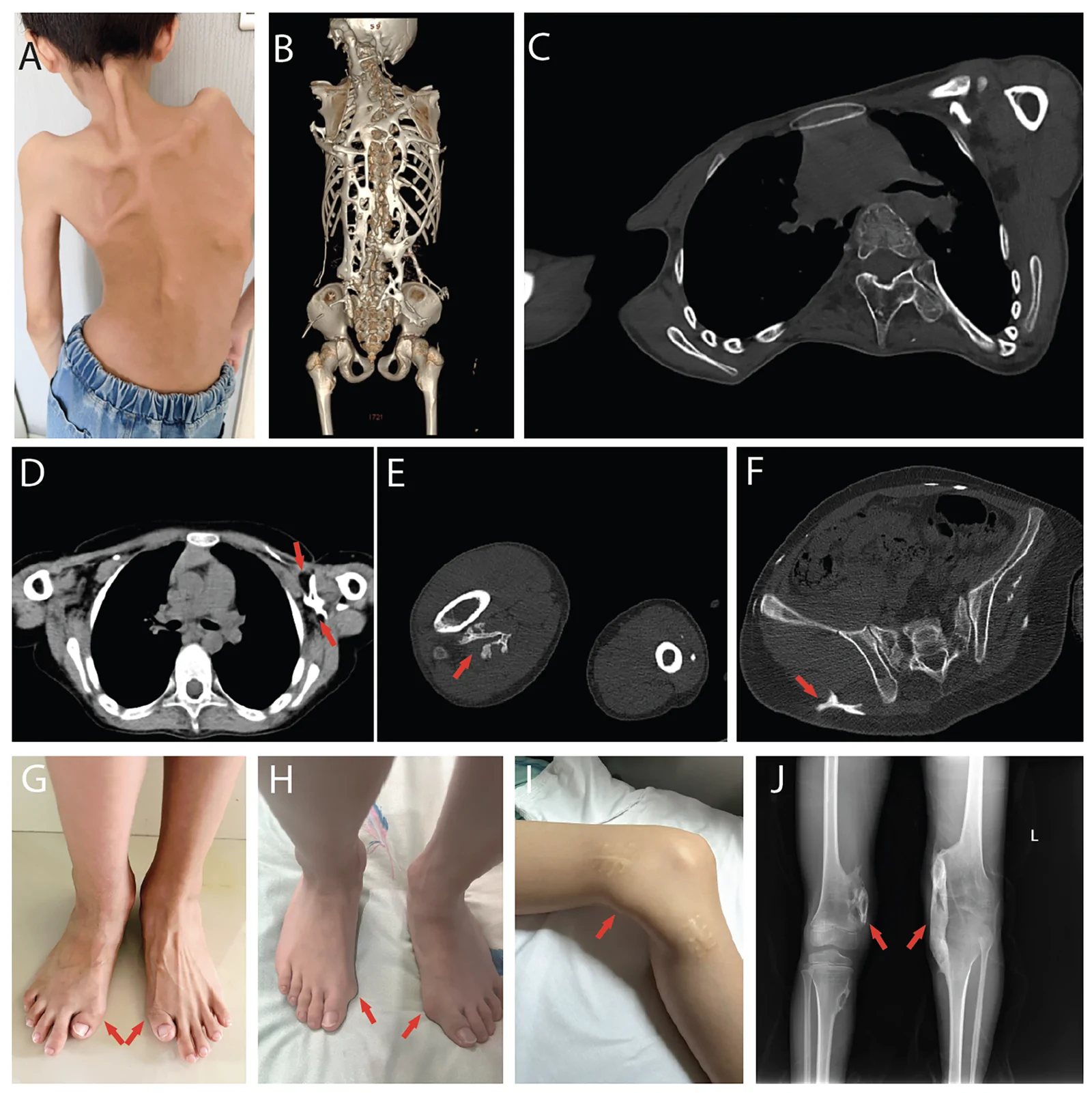
Representative clinical and radiographic manifestations of fibrodysplasia ossificans progressiva (FOP). **(A)** Clinical photograph showing marked subcutaneous heterotopic ossification (HO), shoulder asymmetry and spinal deformity. **(B)** Three-dimensional CT reconstruction demonstrating extensive HO involving the posterior trunk and paraspinal regions. **(C)** Axial CT image showing severe thoracic cage asymmetry and deformity, leading to ventilatory impairment. **(D–F)** CT images demonstrating mature HO within the soft tissues around the shoulder girdle, extremity, and gluteal region, respectively (red arrows). **(G, H)** Congenital bilateral great-toe malformations of FOP, characterized by shortening and lateral deviation of the great toes (red arrows). **(I, J)** Clinical photograph and X-ray radiograph of a patient who underwent inappropriate surgical excision of HO around the left knee, followed by the development of new and more extensive HO at the surgical site and complete immobility of the left knee joint (red arrows).

### Initial presentation and diagnostic pathway

Diagnostic pathway variables are shown in **Table 2**. FOP was suggested at the first hospital visit in 29/114 (25.4%); conversely, 85/114 (74.6%) reported that FOP was not considered at the first visit. The most common first-contact specialties were orthopedics (32/114, 28.1%) and pediatrics (22/114, 19.3%), while departments that most often gave the first FOP diagnosis were rheumatology (33/114, 28.9%) and endocrinology (24/114, 21.1%).

**Table 2:** Diagnostic pathway among 114 patients with FOP.

Domain	Variable	Value
FOP considered at first hospital visit	Yes, n/N (%)	29/114 (25.4)
	No, n/N (%)	85/114 (74.6)
First-contact specialty	Orthopedics, n/N (%)	32/114 (28.1)
	Pediatrics, n/N (%)	22/114 (19.3)
	General surgery/other surgery, n/N (%)	18/114 (15.8)
	Rheumatology, n/N (%)	13/114 (11.4)
	Endocrinology, n/N (%)	10/114 (8.8)
	Other, n/N (%)	19/114 (16.7)
Specialty giving first FOP diagnosis	Rheumatology, n/N (%)	33/114 (28.9)
	Endocrinology, n/N (%)	24/114 (21.1)
	Orthopedics, n/N (%)	18/114 (15.8)
	Pediatrics, n/N (%)	11/114 (9.6)
	General surgery/other surgery, n/N (%)	4/114 (3.5)
	Other/unknown, n/N (%)	24/114 (21.1)

### Misdiagnosis and missed diagnostic opportunities

Total diagnostic delay was evaluable in 113 of 114 patients. 15 of 113 (13.3%) were diagnosed after >1–3 years, 13 of 113 (11.5%) after >3–5 years, 15 of 113 (13.3%) after >5–10 years, and 11 of 113 (9.7%) after >10 years. Overall, 39 of 113 patients (34.5%) had diagnostic delay exceeding 3 years (**Table 3**). Sensitivity analyses of total-delay coding were shown in **Supplementary Table 1**.

**Table 3:** Misdiagnosis, missed diagnostic opportunities, and high-risk procedural exposures.

Domain	Variable	Value
Reported total diagnostic delay	Median years (IQR)	1 (0.3–5); n=113
	≤1 year n/N (%)	59/113 (52.2)
	>1–3 years n/N (%)	15/113 (13.3)
	>3–5 years n/N (%)	13/113 (11.5)
	>5–10 years n/N (%)	15/113 (13.3)
	>10 years n/N (%)	11/113 (9.7)
Symptom-to-first-visit delay^1^	Median years (IQR)	0.0 (0.0–0.2); n=103
First-visit-to-diagnosis delay^2^	Median years (IQR)	0.5 (0.0–3.0); n=106
Misdiagnosis before FOP recognition	Yes n/N (%)	72/114 (63.2)
	No n/N (%)	25/114 (22.0)
	Uncertain n/N (%)	17/114 (14.9)
Misdiagnosis categories among misdiagnosed patients^9^	Tumor-related diagnoses^3^ n/N (%)	36/72 (50.0)
	Orthopedic/congenital diagnoses^4^ n/N (%)	16/72 (22.2)
	Inflammatory/infectious diagnoses^5^ n/N (%)	16/72 (22.2)
	Neurologic/neuromuscular diagnoses^6^ n/N (%)	2/72 (2.8)
	Rheumatologic/autoimmune diagnoses^7^ n/N (%)	1/72 (1.4)
	Trauma-related diagnoses n/N (%)	1/72 (1.4)
	Other/unclear diagnoses^8^ n/N (%)	9/72 (12.5)
High-risk diagnostic/procedural exposures	Bone/soft-tissue biopsy reported n/N (%)	51/114 (44.7)
	Biopsy followed by reported worsening n/N (%)	41/51 (80.4)
	HO lesion excision reported n/N (%)	25/114 (21.9)
	Post-excision recurrence/new lesion at surgical site n/N (%)	22/25 (88.0)

^1,2^ Component diagnostic intervals were calculated only when both required ages were available and followed the expected chronological order. Records in which the first-visit age preceded the reported postnatal symptom-onset age, or the diagnosis age preceded the first-visit age, were treated as missing for the corresponding interval only; reported ages were not imputed or reordered. ^3^Tumor-related diagnoses include fibroma, chondroma, osteochondroma, myofibroma, rhabdomyosarcoma, myofibroblastoma, etc. ^4^Orthopedic/congenital diagnoses include osteoproliferation, torticollis, atlantoaxial subluxation, hallux valgus, cervical ligament calcification, osteophyte, bony bridge, bone cyst, etc. ^5^Inflammatory/infectious diagnoses include myositis, lymphadenitis, synovitis, fasciitis, parotitis, phlebitis, osteomyelitis, etc. ^6^Neurologic/neuromuscular diagnoses include poliomyelitis, cerebral edema, cerebral palsy, etc. ^7^Rheumatologic/autoimmune diagnoses include scleroderma. ^8^Other/unclear diagnoses include hemangioma, edema, cutaneous mucinosis, acquired hemophilia, etc. ^9^Individual reported misdiagnosis categories are not mutually exclusive.

Definite misdiagnosis before FOP recognition was reported by 72/114 (63.2%). Among these 72 patients with reported misdiagnosis, tumor-related diagnoses were most frequent (36/72, 50.0%), followed by orthopedic or congenital diagnoses (16/72, 22.2%), inflammatory or infectious diagnoses (16/72, 22.2%), neurologic or neuromuscular diagnoses (2/72, 2.8%), rheumatologic or autoimmune diagnoses (1/72, 1.4%), trauma-related diagnoses (1/72, 1.4%), and other/unclear diagnoses (9/72, 12.5%). Diagnostic errors are summarized in **Table 3**; **Figure 2A**. A history of biopsy or HO excision was reported by 47/72 patients (65.3%) with reported misdiagnosis and 7/25 patients (28.0%) without reported misdiagnosis (**Supplementary Table 2**). Definite misdiagnosis status was unavailable for 17 participants.

**Figure 2:**
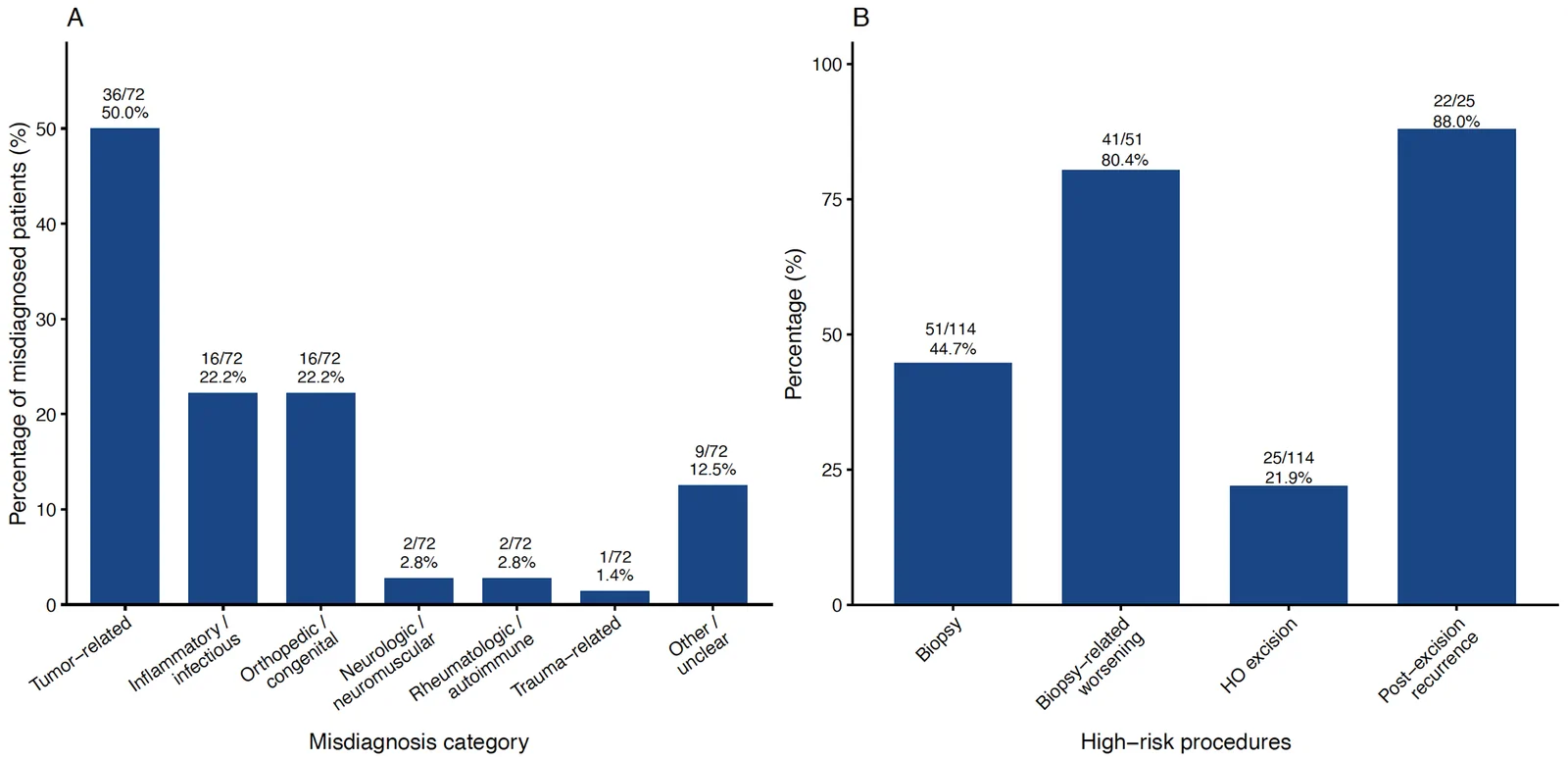
Misdiagnosis categories and high-risk procedure events in the national FOP cohort. **(A)** Distribution of misdiagnosis categories among 72 patients with reported misdiagnosis. Tumor-related diagnoses include fibroma, chondroma, osteochondroma, myofibroma, rhabdomyosarcoma, myofibroblastoma, etc. Orthopedic/congenital diagnoses include osteoproliferation, torticollis, atlantoaxial subluxation, hallux valgus, cervical ligament calcification, osteophyte, bony bridge, bone cyst, etc. Inflammatory/infectious diagnoses include myositis, lymphadenitis, synovitis, fasciitis, parotitis, phlebitis, osteomyelitis, etc. Neurologic/neuromuscular diagnoses include poliomyelitis, cerebral edema, cerebral palsy, etc. Rheumatologic/autoimmune diagnoses include scleroderma. Other/unclear diagnoses include hemangioma, edema, cutaneous mucinosis, acquired hemophilia, etc. Individual reported misdiagnosis categories are not mutually exclusive. **(B)** Percentages of high-risk procedural events, including biopsy, biopsy-related worsening, HO excision and post-excision recurrence. HO, heterotopic ossification.

### Procedures and procedure-associated worsening

Bone or soft-tissue biopsy was reported by 51/114 (44.7%). Among these patients, 41/51 (80.4%) reported subsequent worsening after biopsy. HO lesion excision was reported by 25/114 (21.9%), and 22/25 (88.0%) reported post-excision recurrence or new lesions at the surgical site (**Table 3**; **Figure 2B**). These findings underscore the patient-safety implications of diagnostic error in FOP.

## Discussion

This national cohort demonstrates that diagnostic error remains common and clinically important in FOP. FOP was not considered at the first hospital visit in 74.6% of patients, and 63.2% reported misdiagnosis before recognition of the disorder. In total, 34.5% of patients experienced a delay longer than 3 years and 9.7% waited more than 10 years. Tumor-related, orthopedic or congenital, and inflammatory or infectious conditions accounted for most erroneous diagnoses. Together, these findings identify recurrent failure points at the interface between early clinical presentation, specialty referral, and decisions about invasive investigation.

Our findings extend previous Chinese evidence regarding the diagnosis experience of FOP in this population. Zhang et al. previously characterized the phenotype and genotype of 72 Chinese patients with FOP (20), and Xu et al. subsequently reported prolonged diagnostic delay, frequent misdiagnosis, and disease burden among 67 Chinese patients (21). Some participants in this study may also have been included in earlier Chinese FOP cohorts because these studies drew from the same rare disease population and potentially overlapping clinical networks. Participant-level linkage with the earlier cohorts was unavailable; therefore, the extent of overlap could not be determined. The present study confirms that these problems remain common and further characterizes the diagnostic journey by distinguishing definite misdiagnosis from missed diagnostic opportunities, examining the transition from first-contact specialties to specialties establishing the FOP diagnosis, and reporting procedure-specific outcomes among patients who underwent biopsy or HO excision.

The burden observed in this Chinese national cohort is consistent with the longstanding international experience of diagnostic difficulty in FOP. In the landmark worldwide survey by Kitterman et al., 87% of respondents initially received an incorrect diagnosis, the mean interval to correct diagnosis was 4.1 years, and biopsy was frequently performed (17). International registry studies and national case series have continued to document delayed recognition and heterogeneous diagnostic pathways (25–27). Direct numerical comparisons should be made cautiously because sampling frames, calendar periods, definitions of delay, and reliance on patient report differ across studies. Nevertheless, the persistence of the same broad error patterns suggests that greater access to molecular diagnosis has not eliminated the need for basic clinical recognition.

A central finding is the distinction between misdiagnosis and missed diagnostic opportunity. The former reflects an incorrect label assigned to a local lesion; the latter reflects failure to recognize a syndromic pattern despite available clues. Congenital great-toe malformation was reported by 89.5% of patients. The great-toe phenotype precedes postnatal HO and can be identified by a brief physical examination (11, 14, 28). In a child with recurrent soft-tissue swelling or unexplained restriction of movement, examination of the feet may therefore be more diagnostically useful than an immediate lesion-centered work-up.

Tumor-related diagnoses were the most frequent errors, accounting for half of all misdiagnoses. This pattern is biologically plausible: early FOP lesions may be rapidly enlarging, painful, and highly cellular, while imaging appearance changes as lesions evolve from inflammatory soft-tissue swellings to mature HO (15, 16, 29). The resulting concern for neoplasia can prompt biopsy or excision before the congenital skeletal phenotype is recognized. In our cohort, 44.7% reported biopsy and 80.4% of those patients reported subsequent worsening; 21.9% reported HO lesion excision and 88.0% of those patients reported recurrence or new lesions at the surgical site. These associations are patient-reported and cannot establish causality in each individual case, but they are consistent with the well-described trauma sensitivity of FOP and with expert management recommendations (7, 17, 30).

The distribution of specialties suggests concrete targets for education and referral. Orthopedics and pediatrics were the most common first-contact specialties, whereas rheumatology and endocrinology most often established the first FOP diagnosis. Awareness efforts should therefore extend beyond rare-bone-disease centers to orthopedic and general surgeons, pediatricians, oncologists, radiologists, dentists, and oral-maxillofacial teams. A short red-flag checklist—recurrent swelling, congenital great-toe abnormality, unexplained HO, progressive restriction of movement, and worsening after minor trauma—may be more practical than expecting broad familiarity with an ultra-rare disorder. Multidisciplinary coordination is also important because delayed diagnosis and fragmented care add to the substantial burden experienced by patients and families (31–33).

Based on the observed failure points, we propose a simple early-recognition pathway (**Figure 3**). In a child or young adult with recurrent soft-tissue swelling, unexplained HO, progressive restriction of movement, or a flare-up after minor trauma, clinicians should examine the great toes and thumbs, review congenital skeletal findings and the temporal evolution of imaging, and pause before any invasive procedure. When the phenotype is compatible with FOP, biopsy, excision, intramuscular injection, and forceful manipulation should be avoided; *ACVR1* testing and referral to an experienced FOP or rare-bone-disease center should follow (14, 34, 35). This pathway is intended as a pragmatic safety tool for non-specialists and requires prospective evaluation before its effect on diagnostic delay can be established.

**Figure 3:**
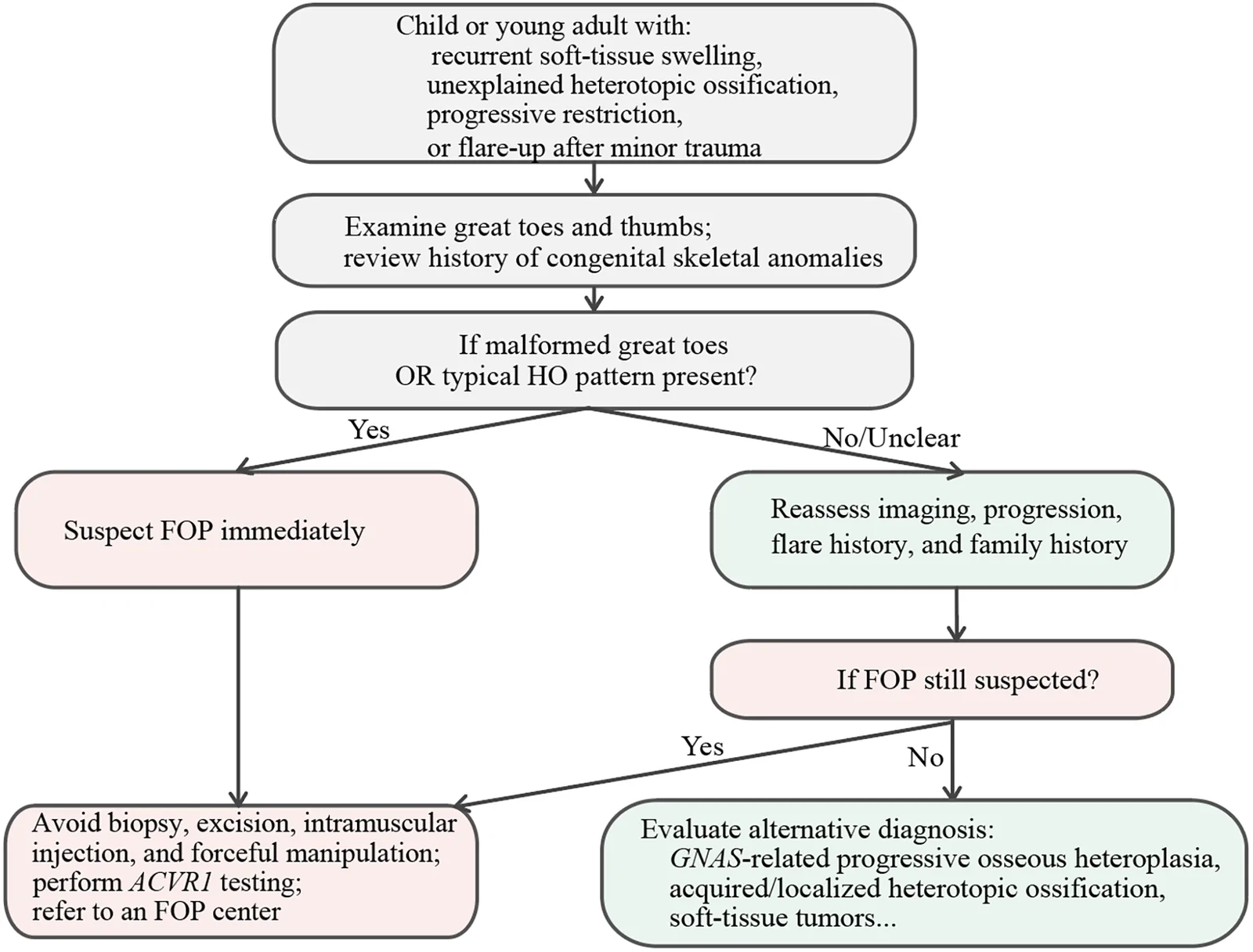
Proposed early-recognition pathway for FOP. Children or young adults with recurrent soft-tissue swelling, unexplained heterotopic ossification (HO), progressive restriction of movement, or flare-up after minor trauma should undergo examination of the great toes and thumbs. Malformed great toes or a typical HO pattern should prompt immediate suspicion of FOP. When these findings are absent or unclear, imaging, disease progression, flare history, and family history should be reassessed. If FOP remains suspected, invasive procedures should be avoided, *ACVR1* testing should be performed, and the patient should be referred to an experienced FOP or rare bone disease center. If FOP is not suspected after reassessment, alternative diagnoses should be evaluated and managed.

This study has several strengths. It evaluates a geographically broad national cohort that is substantial for an ultra-rare disease, focuses specifically on the diagnostic journey, and summarizes misdiagnosis patterns. It also links the diagnostic pathway to patient-reported outcomes after biopsy and HO excision and translates the findings into proposed clinical recognition and referral framework.

Several limitations should be considered. The study was cross-sectional and questionnaire-based, and diagnostic events that occurred years earlier are susceptible to recall error. Free-text responses required normalization and coding, and some responses were missing or unparseable. Original records, imaging, and pathology reports were not uniformly available for independent verification. Biopsy timing and the dates of individual misdiagnosis episodes were not systematically recorded in this dataset, making it infeasible to establish that invasive procedures resulted from diagnostic errors. Worsening after biopsy or surgery was self-reported, and causality and the magnitude of change could not be adjudicated. The registry may preferentially include patients connected to tertiary centers or patient networks and cannot represent individuals who remain undiagnosed. Quality of life was evaluated using a self-rated, non-validated single-item measure. The present cohort was not sufficiently diverse for genotype-stratified analysis, as most patients (111/114, 97.4%) carried the common *ACVR1* c.617G>A (p.Arg206His) variant associated with the classic phenotype, while only three carried non-classic variants.

## Conclusions

Diagnostic error remains common in FOP. In this national cohort of 114 patients, FOP was not considered at the first hospital visit in 74.6% of patients, and 63.2% reported a definite misdiagnosis. Biopsy and HO excision were frequently followed by patient-reported worsening or recurrence. Systematic examination of the great toes, avoidance of invasive procedures when FOP is suspected, early *ACVR1* testing, and timely referral to experienced centers constitute a pragmatic framework for earlier recognition. Whether implementation of this framework shortens diagnostic delay or reduces iatrogenic harm requires prospective validation.

## Data Availability

The raw data supporting the conclusions of this article will be made available by the authors, without undue reservation.
