# Supplementary material for: The diagnostic journey of fibrodysplasia ossificans progressiva: evidence from a national cohort of 114 patients
Source: Front Endocrinol (Lausanne). 2026 Oct 14;17:1941326. doi: 10.3389/fendo.2026.1941326 (PMC13713263; doi:10.3389/fendo.2026.1941326)
Supplement: Supplementary file 1 [file DataSheet1.pdf]

Supplementary Materials of:

The Diagnostic Journey of Fibrodysplasia Ossificans Progressiva: Evidence from a National Cohort of 114 Patients

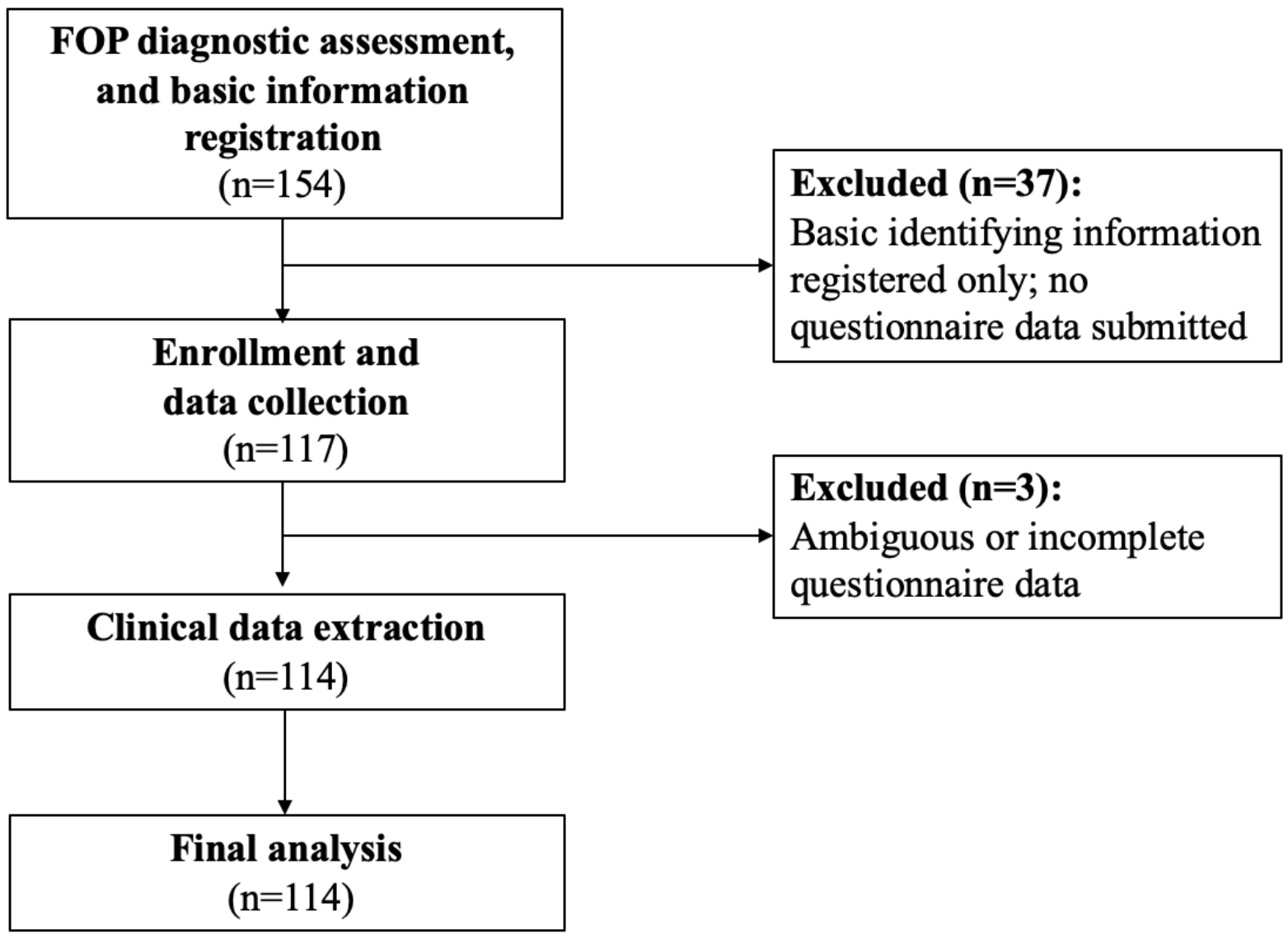

Supplementary Figure 1. Flow diagram of participant inclusion. A total of 154 patients were assessed for a diagnosis of fibrodysplasia ossificans progressiva and have basic information registered. 37 patients were excluded because they didn't have questionnaire data submitted. Three patients were excluded because their questionnaire data were ambiguous or incomplete, leaving 114 patients in the final analysis.

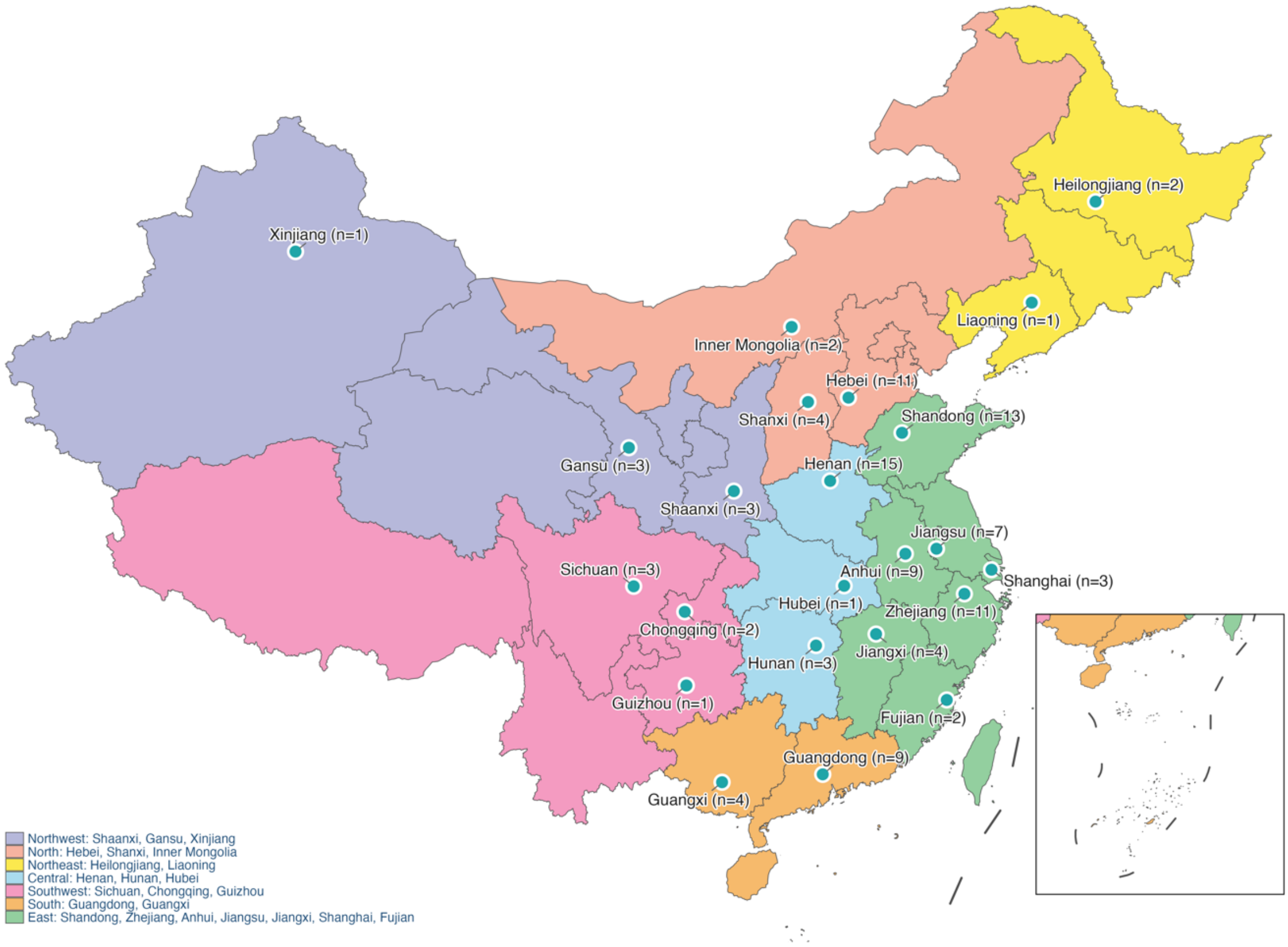

Supplementary Figure 2. Geographic distribution of participants with fibrodysplasia ossificans progressiva in China. The 114 participants included in the final analysis were recruited from 23 provincial-level regions. Map markers and labels indicate the number of participants from each region, and background colors denote the broad geographic divisions used for visualization (Northwest, North, Northeast, Central, Southwest, South, and East).

Supplementary Table 1. Sensitivity of total diagnostic delay to coding of qualified responses

Analysis	n	Median (IQR), years	Delay >3 years, n (%)	Delay >10 years, n (%)
Primary coding	113	1.00 (0.33–5.00)	39 (34.5)	11 (9.7)
All bounded/approximate responses: lower bound	113	1.00 (0.25–5.00)	39 (34.5)	11 (9.7)
All bounded/approximate responses: upper bound	113	1.00 (0.33–5.00)	39 (34.5)	11 (9.7)

Sensitivity analysis included 5 bounded/approximate response of total diagnostic delay: "less than 1 year" (two patients), "less than 1 month" (one patient), "1 year plus" (one patient) and "5 years plus" (one patient). In the primary coding, these were assigned midpoint values of 0.5 year, 0.5/12 year, 1.5 years, and 5.5 years, respectively. The lower-bound scenario assigned values of 0, 0, 1, and 5 years, whereas the upper-bound scenario assigned values of 1, 1/12, 2, and 6 years. One of 114 patients has no evaluable total delay and is excluded from every scenario.

Supplementary Table 2. Reported biopsy and heterotopic ossification excision history by misdiagnosis status.

Reported procedural history	Misdiagnosis: yes (n=72)	Misdiagnosis: no (n=25)	Misdiagnosis: uncertain (n=17)
Biopsy	37 (51.4%)	6 (24.0%)	8 (47.1%)
HO excision	21 (29.2%)	2 (8.0%)	2 (11.8%)
Either biopsy or HO excision	47 (65.3%)	7 (28.0%)	8 (47.1%)
Neither procedure	25 (34.7%)	18 (72.0%)	9 (52.9%)

HO: heterotopic ossification.

Supplementary Information.
Fibrodysplasia Ossificans Progressiva (FOP) Patient Diagnosis & Treatment Journey and Baseline Information Questionnaire
Date completed: ___________
Patient name: ___________

I. Basic Information

1. **Sex:** ☐ Male ☐ Female ☐ Other
2. **Date of birth:** ____ (YYYY) / ____ (MM) / ____ (DD) (Current age: ____ years)
3. **Height:** ____cm **Weight:** ____kg
4. **Current place of residence:** ________ Province, ________ City
5. **Highest level of education:** ☐ Primary school or below ☐ Junior high school ☐ Senior high / secondary vocational school ☐ Junior college ☐ Bachelor's degree ☐ Master's degree or above
6. **Employment status:** ☐ Student ☐ Employed ☐ Unemployed ☐ Retired ☐ Other ________
7. **Type of medical insurance:** ☐ Urban employee basic medical insurance ☐ Urban–rural resident basic medical insurance ☐ Commercial insurance ☐ Publicly funded healthcare ☐ No insurance ☐ Other ________
8. **Annual household income (RMB):** ☐ <50,000 ☐ 50,000–100,000 ☐ 100,000–200,000 ☐ 200,000–500,000 ☐ >500,000 ☐ Prefer not to say
9. Family history: ☐ Other FOP patient(s) in the family — relationship: __________ ☐ None ☐ Don't know

II. Disease Discovery

1. **When and how did you first learn that you might have FOP?**
   ☐ Found during a routine physical examination
   ☐ Found when seeking care for congenital toe/finger malformations
   ☐ Found after seeking care for a localized lump or swelling
   ☐ Found after seeking care for restricted joint movement
   ☐ Found after seeking care for other complaints — please describe: ___________________________
2. **Age at first onset of symptoms or problems possibly related to FOP:** ____________________
3. **Specific symptoms or problems at first onset:** _______________________________________
4. **Before learning that you might have FOP, did you experience any of the following symptoms or signs? (Select all that apply)**
   ☐ Malformed big toes — turned inward or short (present at birth)
   ☐ Restricted neck movement
   ☐ Painful lump on some part of the body
   ☐ Localized skin redness, swelling and warmth
   ☐ Gradual joint stiffness and restricted movement

☐ Hard, painless lump on the shoulder, back or neck

☐ Difficulty opening the mouth

☐ Difficulty breathing

☐ Abnormal walking or gait

☐ Other: _________

5. **Main reason or complaint for the first hospital visit:**

   ____

6. **Hospital of the first visit:**

   ____

7. **Department of the first visit:**

   ____

8. **Age at the first visit: ________________**
9. **Did the hospital at your first visit suggest that you might have FOP?**

   ☐ Yes

   ☐ No

10. **Diagnosis given at the first visit:**

    ____

[Answer only if Q9 is 'No']

III. Medical Consultation History

1. **Date of confirmed FOP diagnosis: ________** (year) **________** (month)
2. **Age at confirmed FOP diagnosis: ________**
3. **Hospital that first gave a definitive FOP diagnosis:**

   ____

4. **Department that first gave a definitive FOP diagnosis:**

   ____

5. **Was there any misdiagnosis before the definitive diagnosis?**

   ☐ Yes

   ☐ No

   ☐ Not sure

6. **If misdiagnosed, the previous (incorrect) diagnoses included:**

   ____

[Answer only if Q5 is 'Yes']

7. **Approximate time from first symptom onset to final diagnosis: _______ years**
8. **Have you ever had any of the following examinations (select all that apply):**

   ☐ CT, X-ray, MRI

   ☐ Whole-body bone scintigraphy / ECT imaging / bone scan

   ☐ Ultrasound of the lesion(s)

   ☐ Genetic testing

   ☐ Bone/soft tissue biopsy

   ☐ Other ____________________

9. **Did the tissue biopsy cause the condition to worsen (including aggravated pain, inflammation, redness, or new onset of flare-ups in surrounding regions after the procedure)?**

☐ Yes (please describe the specific condition) ____________________

☐ No

[Answer only if 'Bone/soft tissue biopsy' was selected in Q8]

10. **Description of the worsening:** __________________

[Answer only if Q9 is 'Yes']

11. **Genetic test result — enter the mutation type (e.g., ACVR1 c.617G>A p.Arg206His) and upload the test report**: ____________________ (please upload the report)

[Answer only if 'Genetic testing' was selected in Q8]

IV. Congenital Features

"Congenital features" refers to abnormalities present at birth, and does not include signs or physical abnormalities that developed later as a result of trauma, surgery, flare-ups or other causes.

1. **Is there a congenital malformation of the big toes?**
   ☐ Yes
   ☐ No
   ☐ Not sure
   ☐ Other
2. **Is there cervical spine fusion (restricted neck movement, with X-ray indicating fusion of cervical vertebrae)?**
   ☐ Yes
   ☐ No
   ☐ Not examined, no obvious signs
3. **Are there any other congenital skeletal abnormalities?**
   ☐ No
   ☐ Yes, please describe: ____________

V. Clinical Manifestations

1. **Has heterotopic ossification occurred to date?**
   ☐ Yes
   ☐ No
   ☐ Not sure
2. **Sites where heterotopic ossification has occurred (select all that apply):**
   ☐ Head/face — describe the exact location and size: ____________
   ☐ Neck — describe the exact location and size: ____________
   ☐ Shoulders — describe the exact location and size: ____________
   ☐ Upper limbs — describe the exact location and size: ____________
   ☐ Hands — describe the exact location and size: ____________
   ☐ Anterior chest — describe the exact location and size: ____________
   ☐ Abdomen — describe the exact location and size: ____________
   ☐ Back — describe the exact location and size: ____________
   ☐ Buttocks — describe the exact location and size: ____________

☐ Lower limbs — describe the exact location and size: ____________

☐ Feet — describe the exact location and size: ____________

[Answer only if Q1 is 'Yes']

3. **Is there currently any restriction of joint movement?**

☐ Yes

☐ No

☐ Not sure

4. **Joints with currently restricted movement (select all that apply):**

☐ Temporomandibular joint (restricted jaw movement)

☐ Cervical spine (restricted looking down / looking up)

☐ Thoracic spine (restricted arching of the back / straightening of the chest)

☐ Lumbar spine (restricted bending and extension of the lower back)

☐ Left shoulder joint

☐ Right shoulder joint

☐ Left elbow joint

☐ Right elbow joint

☐ Left metacarpophalangeal joints

☐ Right metacarpophalangeal joints

☐ Left hip joint (restricted movement of the left thigh)

☐ Right hip joint (restricted movement of the right thigh)

☐ Left knee joint

☐ Right knee joint

☐ Left ankle joint

☐ Right ankle joint

☐ Other: ____________

[Answer only if Q3 is 'Yes']

5. **Is any heterotopic ossification lesion currently painful?**

☐ No

☐ Yes

[Answer only if Q1 is 'Yes']

6. **Do you have difficulty breathing?**

☐ No

☐ Shortness of breath after activity

☐ Shortness of breath at rest

☐ Have had episodes of severe breathlessness

☐ Have depended or currently depend on assisted ventilation (non-invasive ventilator, invasive ventilation, etc.)

7. **Do you have difficulty opening your mouth?**

☐ No

☐ Mildly restricted, able to eat normally

☐ Moderately restricted, unable to eat some foods

☐ Severely restricted, unable to open the mouth more than 1 cm

☐ Completely restricted, the jaw cannot move

8. **Do you have decreased vision / visual field defects?**

   ☐ No

   ☐ Unilateral impairment

   ☐ Bilateral impairment

9. **Do you have hearing loss / deafness?**

   ☐ No

   ☐ Unilateral impairment

   ☐ Bilateral impairment

10. **Do you have any other diagnosed comorbidities (e.g., hypertension, glaucoma, Marfan syndrome)? If so, please list them: ____________**
11. **Other symptoms/clinical manifestations you would like to mention that are not covered above: ____________________________**

VI. Flare-up History

An FOP flare-up refers to the sudden appearance of a painful lump with localized redness and swelling, which may be accompanied by the formation of new heterotopic ossification.

1. **How many flare-ups have you experienced to date?**

   ☐ Never had a definite flare-up

   ☐ 1–3

   ☐ 4–10

   ☐ More than 10

   ☐ Have had definite flare-ups, but cannot count them accurately

2. **Age at first flare-up: _________** years

[Answer only if Q1 is not 'Never had a flare-up']

3. **Average frequency of flare-ups**

   ☐ Less than once a year

   ☐ 1–2 times a year

   ☐ 3 or more times a year

   ☐ Irregular, frequency cannot be estimated

[Answer only if Q1 is not 'Never had a flare-up']

4. **Longest duration of a flare-up**

   ☐ Less than 1 day

   ☐ 1–7 days

   ☐ 1–4 weeks

   ☐ More than 1 month

[Answer only if Q1 is not 'Never had a flare-up']

5. **Symptoms/signs experienced during flare-ups (select all that apply)**

☐ Continuously enlarging local lump

☐ Localized skin redness, swelling and warmth

☐ Persistent low-grade fever

☐ General fatigue

☐ Lump becoming hard and fixed in position

☐ Worsening restriction of joint movement

☐ Other: _________

[Answer only if Q1 is not 'Never had a flare-up']

6. **Did the flare-ups result in new heterotopic bone formation (heterotopic ossification)?**

☐ Every flare-up resulted in ossification

☐ Some resulted in ossification

☐ No confirmed ossification

☐ Never determined

[Answer only if Q1 is not 'Never had a flare-up']

VII. Triggers of Flare-ups and Heterotopic Ossification

1. **Have you ever experienced any of the following events? (Select all that apply)**

☐ Soft tissue injury or muscle strain from a fall, collision, etc.

☐ Fracture or dislocation caused by trauma

☐ Any surgical procedure

☐ Biopsy of soft tissue or an ossified lesion (without resection)

☐ Resection of a heterotopic ossification lesion

☐ Dental procedure without special precautions

☐ Deep intramuscular injection (e.g., vaccination)

☐ None of the above

2. **Did any of the above events lead to an FOP flare-up?**

☐ Yes

☐ No

☐ Not sure

[Answer only if Q1 is not 'None of the above']

3. **Which of the above events led to an FOP flare-up: ___________________**

[Answer only if Q2 is 'Yes']

4. **Are you currently familiar with FOP-related risks and their preventive measures?**

☐ Yes

☐ No

VIII. Treatment History

1. **Have you ever been treated with glucocorticoids during a flare-up?**

☐ No

☐ Yes

Drug name (select all that apply):

☐ Prednisone

Dose and duration: ____________

Treatment effect: ☐ Marked relief of swelling/pain ☐ Slight relief ☐ No effect ☐ Don't know

☐ Methylprednisolone

Dose and duration: ____________

Treatment effect: ☐ Marked relief of swelling/pain ☐ Slight relief ☐ No effect ☐ Don't know

☐ Dexamethasone

Dose and duration: ____________

Treatment effect: ☐ Marked relief of swelling/pain ☐ Slight relief ☐ No effect ☐ Don't know

☐ Other: ____________

Dose and duration: ____________ (if more than one drug, please list each separately)

Treatment effect: ☐ Marked relief of swelling/pain ☐ Slight relief ☐ No effect ☐ Don't know

2. **Have you received, or are you currently receiving, novel/experimental treatment? (Select all that apply)**

☐ No

☐ Previously received experimental treatment, now discontinued

☐ Currently receiving experimental treatment

3. **Type of clinical drug trial received:** ____________________

[Answer only if Q2 is not 'No']

4. **Have you ever undergone surgical treatment (including resection of heterotopic ossification lesions)?**

☐ No

☐ Yes

5. **Please describe the type of surgery, when it was performed, and changes in your condition afterwards:** _________________________

[Answer only if Q4 is 'Yes']

6. **Do you currently use any of the following assistive devices? (Select all that apply)**

☐ No

☐ Crutches / walker

☐ Wheelchair

☐ Orthosis / brace

☐ Respiratory support device

☐ Hearing aid

☐ Other: ________

IX. Disease Progression

1. **Current progression of heterotopic ossification:**

☐ Stable (no obvious new ossification lesions)

☐ Slow progression (occasional new ossification lesions each year)

☐ Rapid progression (multiple new ossification lesions each year)

☐ Don't know

2. **Have any new ossification lesions appeared in the past year:**

☐ No

☐ Yes (site: ________________)

X. Effects of Puberty and Reproduction on the Disease

1. **Disease activity after puberty:**

☐ Markedly reduced

☐ Slightly reduced

☐ No change

☐ Slightly worse

☐ Markedly worse

☐ Not yet reached puberty

[For patients aged ≥ 12 years only]

2. **Have you ever tried to conceive regularly for one year or more (female patients):**

☐ Yes

☐ No

[For female patients aged ≥ 18 years only]

3. **Have you ever been pregnant (female patients):**

☐ Yes

☐ No

[For female patients aged ≥ 18 years only]

4. **Impact of pregnancy/childbirth on FOP (female patients):**

☐ No impact

☐ Worsened (frequent flare-ups or new ossification lesions)

☐ Improved

☐ Don't know

[Answer only if Q3 is 'Yes']

XI. Disease Monitoring and Follow-up

1. **Main hospital currently attended:** ______________________________________
2. **Main department currently attended:** ____________________________________
3. **Main treating doctor:** ______________________________________
4. **Frequency of follow-up visits:**

☐ Monthly

☐ Every 3 months

☐ Every 6 months

☐ Yearly

☐ Irregular

☐ Never

5. **If you have regular follow-up, which items are checked regularly (select all that apply):**

☐ X-ray/CT/MRI

☐ Pulmonary function test

☐ Hearing test

☐ Vision test

☐ Other: ___________

6. **If you do not have regular follow-up, what do you consider the main barriers to regular follow-up (select all that apply):**

☐ Financial burden

☐ Long travel distance

☐ Scheduling conflicts

☐ Do not consider it necessary

☐ Lack of an FOP doctor available for regular follow-up

☐ Other reasons: _________

XII. Quality of Life and Psychosocial Status

1. **Ability to perform daily activities:**

☐ Fully independent

☐ Mostly independent, some help needed

☐ Help needed for most activities

☐ Completely dependent on others

2. **Walking ability:**

☐ Normal

☐ Mildly limited

☐ Requires assistive device

☐ Bedridden

3. **Impact of the disease on study/work:**

☐ No impact

☐ Mild impact, able to continue

☐ Severe impact, intermittent

☐ Forced to stop

4. **Impact of the disease on social activities:**

☐ No impact

☐ Mild impact

☐ Significant impact

☐ Severe impact

5. **Impact of the disease on appearance/posture:**
   ☐ No impact
   ☐ Mild impact
   ☐ Significant impact
   ☐ Severe impact
6. **Do you feel inferior or anxious because of the disease:**
   ☐ Never
   ☐ Occasionally
   ☐ Often
   ☐ Always
7. **Self-rated overall quality of life (0–10, where 0 = very poor and 10 = excellent):**
   ____ points

XIII. Medical Cost Burden

1. **Average annual total medical expenses, RMB (including medical, transportation and accommodation costs):**
   ☐ <10,000
   ☐ 10,000–50,000
   ☐ 50,000–100,000
   ☐ More than 100,000
2. **Impact of medical expenses on household finances:**
   ☐ No burden
   ☐ Mild burden, manageable
   ☐ Considerable burden, affects daily life
   ☐ Extreme burden, unaffordable
3. **Have you ever given up any treatment (including regular follow-up) because of cost:**
   ☐ No
   ☐ Yes (please specify: _________________)

XIV. Baseline Serological and Imaging Data

The items in this section are intended to collect baseline data for disease assessment and are optional. If a test was not performed, the report has been lost, you cannot accurately recall past results, or a question is unclear or difficult to understand, you may skip it. For every item in this section you may photograph or upload the laboratory/examination report; if a report is available, there is no need to fill in the text fields.

1. **Baseline serum marker levels (attachments may be uploaded)**
   Date of testing: _______ (year) _______ (month)
   Aspartate aminotransferase (AST): _______ U/L
   Alanine aminotransferase (ALT): _______ U/L
   Gamma-glutamyl transferase (GGT): _______ U/L
   Alkaline phosphatase (ALP): _______ U/L
   Parathyroid hormone (PTH): _______ pg/mL

Serum 25-hydroxyvitamin D (25OHD): _______ ng/mL
Type I collagen C-terminal telopeptide (β-CTX): _______ ng/mL
Total procollagen type I N-terminal propeptide (P1NP): _______ ng/mL

2. Inflammatory marker levels

High-sensitivity C-reactive protein (hsCRP): _______ mg/L
Erythrocyte sedimentation rate (ESR): _______ mm/h

3. Auxiliary examinations

· **Whole-body bone scintigraphy:** Please upload the most recent whole-body bone scan report (image or file)

· **X-ray:** Please upload the report of the most recent comprehensive X-ray examination (image or file)

· **CT:** Please upload the report of the most recent comprehensive CT examination (image or file)

· **Magnetic resonance imaging (MRI):** Please upload the report of the most recent comprehensive MRI examination (image or file)

· **Pulmonary function:** Please upload the most recent pulmonary function test report (image or file)

· **Pure-tone audiometry:** Please upload the most recent pure-tone audiometry report (image or file)

· **Echocardiography (ECHO):** Please upload the most recent echocardiography report (image or file)

· **Biopsy pathology:** Please upload all biopsy pathology reports (images or files)
